# Protocol for ethyl methanesulphonate (EMS) mutagenesis application in rice

**DOI:** 10.12688/openreseurope.13317.3

**Published:** 2022-02-14

**Authors:** Rasim Unan, Ilyas Deligoz, Kassim Al-Khatib, Husrev Mennan

**Affiliations:** 1 Black Sea Agricultural Research Institute, Samsun, Turkey; 2 University of California, Davis, CA, USA; 3 Ondokuz Mayis University, Samsun, Turkey

**Keywords:** EMS, dose, mutagenesis, protocol, rice

## Abstract

**Background: **Non-transgenic chemical mutagen application, particularly ethyl methanesulfonate (EMS), is an important tool to create mutations and gain a new genetic makeup for plants. It is useful to obtain a sufficient number of mutant plants instead of working with a severe mutation in a few plants. EMS dose and exposure period have been previously studied in several crops; however, EMS used to create point mutations in presoaked rice seeds has not been sufficiently studied and there is no standard protocol for such treatment. The aim of this study is to establish a standard protocol for EMS mutagenesis application in rice.

**Methods: **Two studies were conducted to evaluate the effect of four durations of rice seed presoaking (0, 6, 12, and 24 hours), four EMS concentration doses (0.0%, 0.5%, 1.0%, and 2.0%), and four EMS exposure periods (6, 12, 24, and 48 hours). Germination rate, plumula and radicle length, seedling survival, LD _50 _(Lethal Dose) determination, shoot length, root length and fresh seedling weight were evaluated.

**Results: **Results showed that a 12-hour presoaking duration, 0.5% EMS dose, and six hours of EMS exposure were the best practices for the optimum number of mutant plants.

**Conclusions: **In light of both this study and the literature, a standard application protocol was established. This application protocol, detailed in this article, contains the following guidelines: (1) Presoaking: 12 hours, (2) EMS application: 0.5% dose EMS and six hours, (3) Final washing: six hours, (4) Drying: 72 hours at 38°C. A user-friendly protocol has been presented for utilization by researchers.

## Introduction

Rice is the staple food for nearly half of the world’s population, most of whom live in developing countries. Rice is currently grown in over a hundred countries, which produce 755 million tons of paddy rice ( FAO, 2019). Asian countries, including China, India, Indonesia, Bangladesh, Vietnam, Myanmar, Thailand, Philippines, Japan, Pakistan, Cambodia, South Korea, Nepal, and Sri Lanka, account for 90% of the world’s total rice production. Europe, however, has several important rice producing countries such as Italy, Spain, Greece, Portugal, France, Bulgaria and Turkey. In the European Union, the rice production area is approximately 418,000 hectares, total production is close to three million tons with average yields of 6.8 tons per hectare ( FAO, 2019).

Rice accounts for a third of the earth's area planted with fields crops and it supplies 35–60% calories of nutrition to the world population. People globally consumed more rice than wheat or maize, the other two staple foods. Both developed and developing nations alike grow and consume rice. Of the major staple foods of rice, wheat, and corn, rice is the most crucial food particularly for low- and middle-income nations. Rice is an essential component of complicated cereal product systems that impact issues of worldwide concern, such as food sustainability and security, poverty reduction and protection of social legacy ( Chauhan *et al.*, 2017).

Rice production has some crucial problems such as irrigation scarcity, rice blast disease ( *M. oryzae*), weeds and red rice. Full yield capability has not been realized due to the damage from insects and diseases, while weeds limit rice through rivalry for daylight, water, and supplements. Weed rivalry can bring about complete yield loss ( Al-Khatib *et al.*, 2018; Brim-Deforest *et al.*, 2017; Espino *et al.*, 2018; Gibson *et al.*, 2002). Intensive research to solve some of these problems is being carried out supported by the European Commission. The problems of weeds and red rice is especially a problem in Europe because of their direct production system of sowing rice. The main rice area, Asia, has a production system of transplanting rice so they have no severe weed problems in their fields. Therefore, chemical companies have not been willing to develop new active ingredients for European countries. Old herbicides do not work effectively over time. The development of herbicide-tolerant rice is a more reasonable approach than developing a new active ingredient. Researchers have developed herbicide resistance systems such as Clearfield, Provisia, and Roxy Rice by mutation application ( Croughan, 2015; Mankin *et al*., 2014). Most of this research is based on plant EMS mutagenesis application.

Rice plant breeders have used point mutations in their breeding program to overcome these problems. The mutation may exist in nature besides the artificially induced mutation. Physical and chemical mutagens are used to obtain plants by mutation breeding, such as gamma rays, X-rays, fast neutrons and also ethyl methanesulphonate (EMS; CH _3_SO _3_C _2_H _5_), diepoxybutane (DEB, C _4_H _6_O _2_) and sodium azide (NaN _3_) ( FAO/IAEA, 2018). The chemical mutagen EMS has been widely utilized to induce a large number of functional variations in rice. EMS alkylates guanine bases and leads to mispairing of alkylated G with T instead of C, resulting in primarily G/C- to -A/T transitions ( Bhat *et al.*, 2007).

Chemical mutagen application methods have a draft protocol of presoaking, mutagen application and a final washing process. The implementation phase of these processes differs in many studies and unfortunately, there is no standard protocol for mutagenizing rice seeds. The objective of this study is to develop a standard protocol for EMS mutagenesis application in rice.

## Methods

### Materials

*Osmancik-97* is a *Japonica* type Turkish rice variety ( Unan, 2021c). The variety was released by Trakya Agricultural Research Institute, Edirne, Turkey in 1997. The parents are *Rocca* and *Europe*, which originate from Italy. The *Osmancik-97* rice variety has a plant length of 105 cm, 85 days of flowering, 135 days of maturity, a semi horizontal 16 cm panicle, 65% milling yield and 8-9 tons per hectare grain yield potential. Material samples have 14% moisture content, 98-100% germination ratio, 24 g milled 1000 grain and 34 g un-milled 1000 grain weight ( Unan *et al.*, 2013).

The molecular formula of EMS (Sigma- Aldrich Inc., USA) is C _3_H _8_O _3_S, molecular weight is 124.2 g, density is 1.206 g ml ^-1^, half-life is 48.5 hours at 25°C. It is a powerful mutagen for plants.

### EMS mutagenesis

The experiment was carried out using a randomized parcel design with three replications for the germination experiment and four replications for the seedling experiment, and each replication used 100 seeds under a fume hood in a phytotron growth chamber. Seeds were sterilized with bleach solution (30% commercial bleach + 0.02% Triton X-100) for 15 min and washed three times with pure water. Seeds were placed in a glass container and pure water was added to a volume of 1 ml seed ^-1^. Seeds were presoaked for 0, 6, 12 or 24 hours at 20°C. Afterwards, the water was decanted and again 1 ml seed ^-1^ of 0.0%, 0.5%, 1%, or 2% concentrations of EMS (v/v) in water was added. Seeds were incubated for six, 12, 24, or 48 hours in different concentrations of EMS solution at 20°C under the fume hood. Subsequently, EMS-treated seeds were washed with pure water five times for five minutes (total 25 minutes) ( Talebi *et al.*, 2012). The seeds were washed again with running tap water for six hours ( Sagel *et al*., 2017). EMS is a mutagenic chemical and it is important to reduce harmful effects of EMS on the ecology and person health subject to appropriate storage, correct using, suitable disposal and transportation.

Seedling survival rate is the ratio of surviving seedlings 21 days after sowing of seeds ( Evangelina *et al.*, 2010). In the seedling experiment, seedling survival of rice seeds with each of the four presoaking durations, four EMS doses and four exposure periods was determined as the percentage of seedlings that survived 21 days after seeding in the phytotron chamber.

 Seedling survival (%) = (survived rice seedlings / sowed rice seeds) × 100

Imbibition rate was calculated as the percentage of water intake of seeds hourly. 100g of seeds which had 14% water content were incubated in pure water at 20°C and the weight noted each hour for 48 hours with three replications. The seeds were removed from the water, drained for one minute and dried with blotting paper for 30 seconds and then measured with an analytical balance (AS 3Y, Radwag Wagi Elektroniczne, Poland). Imbibition rate was calculated using the following formula:

Imbibition rate (%) = (last weight - first weight) × 100 / first weight

### Experiment 1: Germination experiment

The experiment was carried out using a randomized block design with three replications for germination. Experiment factors were four presoaking durations, four EMS doses, and four EMS exposure periods. 100 EMS-treated seeds were used for each treatment besides 100 untreated control seeds on filter paper soaked in 30ml of pure water in petri dishes. Untreated control seeds were managed under the same conditions except EMS exposure. The seeds were then put in the phytotron at 25°C and 30°C with 12-hour cycles of light and dark conditions for seven days. After seven days, the number of seeds that germinated, with 5 mm plumula being accepted as germinated ( Cruz & Milach, 2004), under these conditions was recorded. Seedling length of the plants were measured using a digital caliper (Insize standart calipper, Germany). The roots were scanned using an Epson 11000XL scanner at a resolution of 600 dpi. Root traits were obtained using WinRHIZO 2009 Pro software (Regent Instruments). The equation to calculate germination percentage was (seeds germinated / total seeds) × 100 ( IRRI, 2002).

### Experiment 2: Seedling experiment

The experiment was carried out using a randomized block design with four replications. Experiment factors are four presoaking durations, four EMS doses, four EMS exposure periods, and their controls. Twenty seeds for each presoaking duration, EMS-treatment and EMS exposure duration seeds and their controls were sown in a plastic plant tray. Control seeds included no-presoaked seeds and no-EMS exposure seeds. Sterilized soil was used in the experiment. The 28-cell plant tray had a diameter of 7 cm and a depth of 7.4 cm. The plant trays were then put in the phytotron at 25°C and 30°C with 12-hour dark and 12-hour light cycles for 21 days, respectively. After 21 days the surviving seedlings’ length, root length and fresh plant weight were measured ( IRRI, 2002). The fresh plant weight measurement equipment used for analytical weighing was manufactured by Radwag Wagi Elektroniczne, Poland (Radwag, AS 3Y analytical balances). The length of the plants was measured using a digital caliper (Insize standart calipper, Germany). The roots were scanned using an Epson 11000XL scanner at a resolution of 600 dpi. Root traits were obtained using WinRHIZO 2009 Pro software (Regent Instruments).

### EMS LD50 Determination

The calculations are based on the following formula according to Spearman - Karger (1931) method:

                LD _50_ = D _h_ – [∑ (a × b) / m]

LD _50_ = Arithmetic means of dose that half of the plant’s dead; D _h_ = highest dose for plants; a = half the sum of the plants reacting with two consecutive doses; b = Mean mortality of the plants between two consecutive doses; m = number of died plants in each group.

### Factsheet and flowchart of protocol for EMS mutagenesis application in rice

A one-page user protocol might be useful in laboratory studies. Hence, a single page user protocol has been created. The materials used in the protocol are simply defined in the factsheet. Protocol application stages and durations are given for presoaking, EMS application, final washing, and drying. In addition, a flowchart is supplied for users. This flowchart shows a schematic illustration for how to utilize the protocol. The factsheet and flowchart of the protocol for EMS mutagenesis application in rice are supplied as *Extended data* ( Unan, 2021b).

### Statistical analysis

Three-way analysis of variance was used in order to detect any statistically significant differences between presoaking duration, EMS dose, and EMS exposure period. Significant differences between the averages were evaluated using the Tukey least significant difference (LSD) test at p-value <0.01. LSD tested the differences in observed averages of all tested parameters between treatment and non-treatment seeds. Statistical analysis was conducted using JMP 7.0 software.

## Results

### Imbibition rate

**Figure 1.**
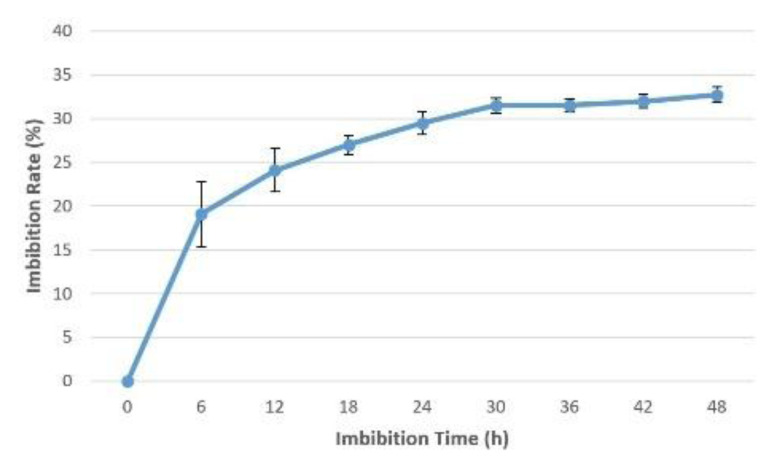
Water uptake measurement compared to imbibition time interval at six hours in *Osmancik-97* rice variety.

**Figure 2.**
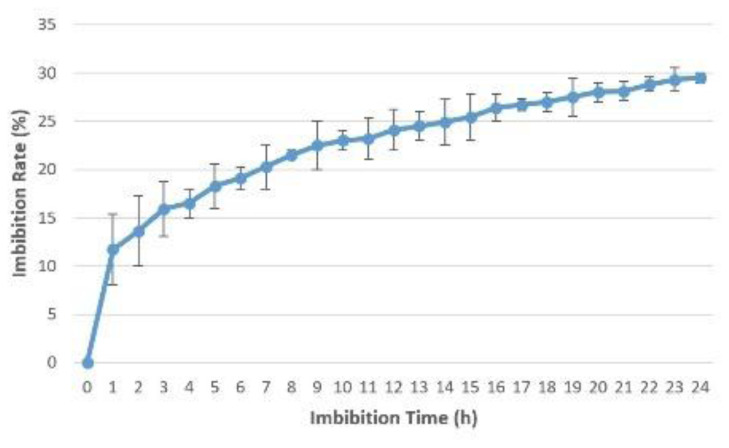
Water uptake measurement compared to imbibition time hourly in *Osmancik-97* rice variety.

The imbibition rate was calculated for *Osmancik-97* rice at the start of the experiment. The increase in seed weight happening over the imbibition time period hourly and every six hours at 20°C in the phytotron was determined ( Figure 1 and Figure 2). Initial moisture content was 14%. The seeds with 14% moisture were considered to have 0% water intake; water intake was calculated as a percentage increase in moisture content. Rapid increases of water uptake were calculated in first hour as more than 10%. Subsequently, the rapid rising proceeded up to 25% in the first 12 hours. Finally, the increase reached 30% in the first 24 hours. No significant increase was seen after 24 hours. The seeds weight reached equilibrium as around 30% in the pure water. During the 0, 6, 12 and 24 hours presoaking (imbibition) stage, the seeds had 0%, 19.1%, 24.1%, and 29.5% water intake, respectively ( Unan, 2021a).

### Germination experiment

Germination is a crucial factor for EMS mutagenesis experiments. The analysis of variance revealed significant (P < 0.01) differences in germination percentage between each EMS dose, exposure period and presoaking and their combinations. Germination was ranked from 0% to 100% in this study. When evaluated in terms of the EMS dose, the lowest average germination observed was 33.4% for the 2% EMS dose. The highest mean germination observed was 98.8% for the control plot ( Table 1). As per Table 1, the outcomes acquired show that a decrease in seed germination occurred with a corresponding increase in EMS dose (P < 0.01). Considering EMS exposure period compared to all controls (0.0%), mean germination percentage was 91.0%, 75.9%, 13.9%, and 0.0% for six, 12, 24, and 48-hour exposure periods, respectively. When evaluated in terms of presoaking, the lowest mean germination (49.1%) for zero hours (dry seed) presoaking. The highest mean germination observed was 71.8% at 12 hours presoaking. Higher dosages EMS application without presoaking prevented germination and all 48-hour durations prevented germination. It should be emphasized that the chemical reduces the germination ability of dry seeds and also EMS application for more than 24 hours prevents germination to a high extent. Most combinations resulted in 100% germination. However, six hours application, 0.5% EMS dose, and 12 hours presoaking interaction might be preferred for maximum germination of mutant seeds.

**Table 1.** Effect of EMS application dose, EMS exposure period and presoaking duration on germination in rice (%).

EMS application dose (%)	EMS exposure period (h)	Presoaking duration (hours)				Mean
		0	6	12	24	
0.0%	6	100a	100a	95b	95b	97.5 c
	12	100a	100a	100a	100a	100.0 a
	24	100a	95b	100a	100a	98.8 b
	48	100a	100	95b	100a	98.8 b
Mean		*100a*	*98.8b*	*97.5c*	*98.8b*	98.8 A
0.5%	6	100a	100a	100a	100a	100.0 a
	12	75e	95b	100a	100a	92.5 d
	24	80d	100a	100a	55g	83.8 f
	48	0l	0l	0l	0l	0.0 k
Mean		*63.g*	*73.8e*	*75.0d*	*63.8g*	69.1 B
1%	6	60f	100a	100a	100a	90.0 e
	12	35i	100a	100a	100a	83.8 f
	24	0l	75e	50h	15j	35.0 i
	48	0l	0l	0l	0l	0.0 k
Mean		*23.8m*	*68.8f*	*62.5h*	*53.8i*	52.2 C
2%	6	35i	100a	100a	85c	80.0 g
	12	0l	15j	100a	80d	48.8 h
	24	0l	10k	10k	0l	5.0 j
	48	0l	0l	0l	0l	0.0 k
Mean		*8.8n*	*31.3l*	*52.5j*	*41.3k*	33.4 D
General Average		49.1D	68.1B	71.8A	64.4C	63
**	**	**				

**: significant at the 1% level; NS: no significant differences. Values followed by the same letter are not statistically significantly different. A: Presoaking duration; A: EMS application dose; a: EMS application dose × EMS exposure period interaction; a: EMS application dose × Presoaking duration; a: Three-way interaction which EMS application Dose × EMS exposure period × Presoaking duration. LSD _dose_ = 0,09; LSD _Duration_= 0.09; LSD _Presoaking_ = 0.11; LSD _Dose×Duration_=0.22; LSD _Dose×Presoaking_=0.22; LSD _Duration×Presoaking_=0.22; LSD _Dose×EMSduration×Presoaking _= 0,45; CV (%) = 4.44. CV, coefficient of variation; EMS, ethyl methanesulfonate; LSD, least significant difference.

Plumula length is another indicator factor used in EMS mutagenesis experiments. There are significant (P < 0.01) differences in plumula length with each EMS dose, exposure period, presoaking, and their combinations according to the analysis of variance. Plumula length ranged from 0 mm to 62.0 mm in the germination experiment. In terms of the EMS dose, the lowest mean plumula length measured was 20.0 mm for the 2% EMS dose plot. The highest mean plumula length measured was 52.6 mm for the control plot ( Table 2). Statistical analysis on plumula length showed an attendant decrease in plumula length with applied increases in the concentration of EMS. As per Table 2, the outcomes acquired show that a decrease in plumula length was observed with a corresponding increase in EMS dose (P < 0.01). When evaluated in terms of the exposure period, the mean plumula length was 50.1, 41.8, 29.6, and 13.0 mm for the six, 12, 24, and 48 hours exposure periods, respectively. Regarding presoaking, the lowest mean plumula length observed was 27.1 mm for the zero hours (dry seed) presoaking plot. The highest mean plumula length observed was 37.5 mm for the 12 hours presoaking plot. EMS application without presoaking and 48 hours of EMS application and their combinations nearly forestalled plumula length. It should be underlined that the EMS harms the plumula length capacity of dry seeds and furthermore EMS application for over 24 hours with EMS application doses of 1–2% forestalls plumula length. Many of the combinations had a 5 mm plumula length. However, six hours application, 0.5% EMS dose, and 24 hours presoaking showed the best results except for the 0% (control) EMS dose application.

**Table 2.** Effect of EMS application dose, EMS exposure period and presoaking duration on plumula length in rice (mm).

EMS application dose (%)	EMS exposure period (h)	Presoaking duration (hours)				Mean
		0	6	12	24	
0.0%	6	62.2ab	60.1ac	49.7hl	52.9ej	56.2 a
	12	50.6fl	50.5fl	51.1fl	51.8fk	51.0 b
	24	47.5ln	52.9ej	49.5hl	54.6df	51.1 b
	48	58.1bd	63.0a	54.0dg	32.9t	52.0 b
Mean		*54.6a*	*56.6a*	*51.1b*	*48.1c*	52.6 A
0.5%	6	42.4oq	53.4ei	49.4hl	57.1ce	50.6 b
	12	31.3t	51.2fl	50.6fl	52.2fk	46.3 c
	24	38.1rs	47.0ln	49.8gl	38.6qs	43.4 d
	48	0.0w	0.0w	0.0w	0.0w	0.0 h
Mean		*27.9f*	*37.9d*	*37.5d*	*36.9d*	35.1 B
1%	6	18.0u	49.1jm	54.1df	53.6eh	43.7 d
	12	40.3pr	44.9mo	47.3ln	43.4np	43.9 d
	24	0.0w	42.6oq	34.8st	7.6v	21.2 f
	48	0.0w	0.0w	0.0w	0.0w	0.0 h
Mean		*14.6h*	*34.1e*	*34.0e*	*26.2f*	27.2 C
2%	6	44.9mo	59.8ac	53.3ei	48.1km	51.5 b
	12	0.0w	15.3u	49.3il	39.5pr	26.0 e
	24	0.0w	4.3v	6.8v	0.0w	2.8 g
	48	0.0w	0.0w	0.0w	0.0w	0.0 h
Mean		*11.2i*	*19.9g*	*27.3f*	*21.9g*	20.0 D
General Average		27.1C	37.1A	37.5A	33.3B	33.7
**	**	**				

**: significant at the 1% level; NS: no significant differences. Values followed by the same letter are not statistically significantly different. A: Presoaking duration; A: EMS application dose; a: EMS application dose × EMS exposure period interaction; a: EMS application dose × Presoaking duration; a: Three-way interaction which EMS application Dose × EMS exposure period × Presoaking duration. LSD _dose_ = 1.0; LSD _Duration_= 1.0; LSD _Presoaking_ = 1.0; LSD _Dose×Duration_=2.1; LSD _Dose×Presoaking_=2.1; LSD _Duration×Presoaking_=2.1; LSD _Dose×EMSduration×Presoaking _= 4.2; CV (%) = 7.7. CV, coefficient of variation; EMS, ethyl methanesulfonate; LSD, least significant difference.

Radicle length ranged from 0.0 to 47.8 mm in this study. The analysis of variance showed significant (P < 0.01) differences in radicle length with each presoaking duration, EMS dose, exposure period, and their combinations. When evaluated in terms of the EMS dose, the lowest and highest mean radicle length observed was 10.7 mm and 33.7 mm for the 2% EMS dose plot and control plot, respectively ( Table 3). Increasing EMS doses caused shortening of the radicle length. Considering each exposure period, the mean radicle length was 33.7, 19.8, 14.9, and 10.7 mm for the six, 12, 24, and 48 hours exposure periods, respectively. When evaluated in terms of presoaking, the lowest and highest mean radicle length observed was 14.9 mm and 23.8 mm for the zero hours (dry seed) and 12 hours presoaking plot, respectively. Many of the combinations resulted in 20 mm radicle length, which is the optimum radicle length. However, 12 hours application, 0.5% EMS dose, and 24 hours presoaking combinations showed the best results except for the 0% (control) EMS dose application.

**Table 3.** Effect of EMS application dose, EMS exposure period and presoaking duration on radicle length in rice (mm).

EMS application dose (%)	EMS exposure period(hour)	Presoaking duration (hours)				Mean
		0	6	12	24	
0.0%	6	25.7jk	31.7fg	43c	46.2ab	36.7 b
	12	42.8c	43.9bc	47.8a	33.3eg	41.9 a
	24	30.8gh	32.1eh	38.4d	29.6hi	32.7 c
	48	32.5eh	29.5hi	21.4lm	11.1o	23.6 f
Mean		*32.9b*	*34.3b*	*37.7a*	*30.1c*	33.7 A
0.5%	6	13.6no	27.5ij	34.ef	35.1e	27.5 e
	12	15.2n	29.6hi	42.0c	46.ab3	33.3 c
	24	19.0m	19.6m	15.1n	12.9no	16.7 h
	48	0.0r	0.0r	0.0r	0.0r	0.0 k
Mean		*11.9ij*	*19.2f*	*22.7d*	*23.5d*	19.8 B
1%	6	10.7o	15.4n	32.0fg	29.7hi	21.9 g
	12	23.kl	34.2ef	32.3eh	19.6m	27.3 e
	24	0.0r	20.0m	18.8m	3.7pq	10.6 j
	48	0.0r	0.0r	0.0r	0.0r	0.0 k
Mean		*8.4k*	*17.4g*	*20.8e*	*13.2hi*	14.9 C
2%	6	25.1jk	34.7ef	34.1ef	23.1kl	29.3 d
	12	0.0r	6.7p	18.6m	24.3kl	12.4 i
	24	0.0r	1.7qr	3.5q	0.0r	1.3 k
	48	0.0r	0.0r	0.0r	0.0r	0.0 k
Mean		*6.3l*	*10.7j*	*14.1h*	*11.9ij*	10.7 D
General average		14.9C	20.4B	23.8A	19.9B	19.7
**	**	**				

**: significant at the 1% level; NS: no significant differences. Values followed by the same letter are not statistically significantly different. A: Presoaking duration; A: EMS application dose; a: EMS application dose × EMS exposure period interaction; a: EMS application dose × Presoaking duration; a: Three-way interaction which EMS application Dose × EMS exposure period × Presoaking duration. LSD _dose_ = 0.7; LSD _Duration_= 0.7; LSD _Presoaking_ = 0.7; LSD _Dose×Duration_=1.5; LSD _Dose×Presoaking_=1.5; LSD _Duration×Presoaking_=1.5; LSD _Dose×EMSduration×Presoaking _= 3.0; CV (%) = 9.4. CV, coefficient of variation; EMS, ethyl methanesulfonate; LSD, least significant difference.

### Seedling experiment

Germinated seeds might lose their vitality over time at the seedling stage. Hence, seedling survival is a crucial factor for mutation experiments. In this study, the germination rate was 98.8% in the germination experiment and survival seedling rate was determined as 90.2% in the seedling experiment in the control plots. Although all conditions and applications are the same, a loss of 8.6% was experienced. This illustrates the importance of seedling trials in addition to germination trials in mutation experiments.

Seedling survival decreased substantially with increasing EMS dose ( Table 4). To investigate the reasons behind this dramatic decrease in seedling survival with increasing EMS dose, the level of seedling damage by EMS exposure period in presoaked and dry seeds before sowing was examined. The presoaking of seeds before sowing has a strong effect on seedling survival rate. This may suggest that presoaked seeds could tolerate EMS exposure periods up to 24 hours, as they tolerate high EMS doses during the seedling stage.

**Table 4.** Effect of EMS application dose, EMS exposure period and presoaking duration on surviving seedling in rice seedling experiment (%).

EMS application dose (%)	EMS exposure period (hours)	Presoaking duration (hours)				Mean
		0	6	12	24	
0.0%	6	68.8bc	93.8a	56.3ce	31.3fh	62.5 c
	12	100.0a	93.8a	100.0a	100.0a	98.4 a
	24	100.0a	100.0a	100.0a	100.0a	100.0 a
	48	100.0a	100.0a	100.0a	100.0a	100.0 a
Mean		*92.1ab*	*96.9a*	*89.1ab*	*82.8b*	90.2 A
0.5%	6	18.8hj	31.3fh	37.5eh	25.0gi	28.1 f
	12	6.3ij	18.8hj	56.3ce	56.3ce	34.4 ef
	24	62.5bd	93.8a	100.0a	62.5bd	79.7 b
	48	0.0j	0.0j	0.0j	0.0j	0.0 i
Mean		*21.9fg*	*35.9d*	*48.4c*	*35.9d*	35.5 B
1%	6	0.0j	43.8dg	81.3ab	62.5bd	46.9 d
	12	0.0j	68.8bc	50.0cf	56.3ce	43.8 de
	24	0.0j	25.0gi	68.8bc	0.0j	23.4 fg
	48	0.0j	0.0j	0.0j	0.0j	0.0 i
Mean		*0.0i*	*34.4de*	*50.0c*	*29.7df*	28.5 C
2%	6	25.0gi	43.8dg	50.0cf	43.8dg	40.6 de
	12	0.0j	0.0j	25.0gi	25.0gi	12.5 gh
	24	0.0j	6.3ij	18.8hj	0.0j	6.25hi
	48	0.0j	0.0j	0.0j	0.0j	0.0 I
Mean		*6.25hi*	*12.5gh*	*23.4eg*	*17.2gh*	14.8 D
General average		30.1C	44.9B	52.7A	41.4B	42.3
**	**	**				

**: significant at the 1% level; NS: no significant differences. Values followed by the same letter are not statistically significantly different. A: Presoaking duration; A: EMS application dose; a: EMS application dose × EMS exposure period interaction; a: EMS application dose × Presoaking duration; a: Three-way interaction which EMS application Dose × EMS exposure period × Presoaking duration. LSD _dose_ = 5.7; LSD _Duration_= 5.7; LSD _Presoaking_ = 5.7; LSD _Dose×Duration_=11.42; LSD _Dose×Presoaking_=11.42; LSD _Duration×Presoaking_=11.42; LSD _Dose×EMSduration×Presoaking _= 23.0; CV (%) = 3.9. CV, coefficient of variation; EMS, ethyl methanesulfonate; LSD, least significant difference.

A significant interaction was also observed between the EMS exposure period and EMS dose. This is might be a result of EMS concentration in seeds increasing with increasing exposure time, particularly when the seeds are incubated in EMS solution for longer.

The analysis of variance revealed significant (P < 0.01) differences in surviving seedlings with each EMS dose, exposure period, presoaking period, and their interactions. The surviving rate was ranked from 0% to 100% in this study. When evaluated in terms of the EMS dose, the lowest survival rate observed was 14.8% for the 2% EMS dose plot. The highest surviving rate observed was 90.2% for the control plot ( Table 4). Statistical analysis on survival rate showed an attendant decrease in germination with applied increases in the concentration of EMS. As per Table 4, the outcomes acquired show that a decrease in seed germination occurred with a corresponding increase in EMS dose (P < 0.01). Considering each exposure period, the mean germination percentage was 44.5%, 47.3%, 52.3%, and 25.0% for 6-, 12-, 24-, and 48-hour exposure periods, respectively. When evaluated in terms of presoaking, the lowest mean survival rate observed was 30.1% for the zero hours (dry seed) presoaking plot. The highest mean survival rate observed was 52.7% for the 12 hours presoaking plot. EMS application without presoaking and 48 hours of EMS application and their combinations almost prevented seedling survival. It should be emphasized that the chemical reduces the germination ability of dry seeds and also EMS application for 48 hours inhibit germination to a high extent. Correspondingly, the survival rate also decreased. In addition, there was a difference between germination rate and survival rate up to 8.6%. It could be reasoned that seedlings that germinated weakly after the mutation application were unable to survive.

There were significant effects of presoaking duration, EMS exposure period, EMS dose, and some of the combinations provided a 100% survival rate. Survival rates were similar for 24-hour exposure period, 0.5% EMS dose and 12 hours presoaking plots compared with control plots.

Seedling shoot length is an important feature showing the development of seedlings after mutation application. Seedling shoot length was significantly (P < 0.01) affected by presoaking duration, EMS dose, EMS exposure period, and their combinations. Seedling shoot length varied between 0.0-36.3 mm and the experiment average was 16.0 mm. The highest mean shoot length was measured 30.6 mm on the control plot ( Table 5). The consequences of the seedling experiment indicated that increasing EMS doses caused a significant decrease in seedling shoot development ( Table 5). A significant decrease was observed of over 50% when EMS dose was 0.5% and higher. As EMS exposure period increased, a significant decrease in seedling shoot length occurred, especially for the 24-hour EMS exposure period. At a dose of 0.5% EMS, the lowest EMS dose, an exposure period of 48 hours resulted in a significant decrease (no growth of shoots) in seedling shoot length compared with the control. The results indicated that no presoaking caused a significant decrease in seedling shoot development. A significant decrease was observed of approximately 50% in the non-presoaked plot. In terms of the interaction between presoaking duration and EMS exposure period, a significant decrease in seedling shoot length occurred, especially for no presoaking and 48-hour EMS exposure period. The longest seedling shoots were observed for the 12 hours presoaking duration, 0.5% EMS dose and 12-hour exposure period conditions when compared to the other combinations except for 0% EMS dose.

**Table 5.** Effect of EMS application dose, EMS exposure period and presoaking duration on shoot length in rice seedling experiment (mm).

EMS application dose (%)	EMS exposure period (h)	Presoaking duration (h)				Mean
		0	6	12	24	
0.0%	6	31.9ae	30.5af	26.5bj	19.3im	27.0 b
	12	33.1ac	31.5af	33.1ac	31.8af	32.4 a
	24	32.6ae	31.7af	33.9ab	30.2ag	32.1 a
	48	21.4hl	36.3a	33.7ab	33.1ad	31.1 ab
Mean		*29.7a*	*32.5a*	*31.8a*	*28.6a*	30.6 A
0.5%	6	18.8jm	17.4ko	24.9ck	18.9jm	19.9 c
	12	4.3pq	12.4mp	30.0ag	26.2bj	18.2 cd
	24	20.7hm	19.6im	24.6ek	18.2jn	20.8 c
	48	0.0q	0.0q	0.0q	0.0q	0.0 f
Mean		*10.9de*	*12.3ce*	*19.9b*	*15.8bc*	14.7 B
1%	6	0.0q	22.0gl	27.6bi	26.3bj	18.9 cd
	12	0.0q	23.5fl	18.2jn	20.3hm	15.5 d
	24	0.0q	9.9np	17.6kn	0.0q	6.9 e
	48	0.0q	0.0q	0.0q	0.0q	0.0 f
Mean		*0.0f*	*13.8cd*	*15.8bc*	*11.6ce*	10.3 C
2%	6	10.3np	28.3ah	16.0lo	24.6dk	19.8 c
	12	0.0q	0.0q	9.1op	25.5bk	8.6 e
	24	0.0q	4.3pq	16.0lo	0.0q	5.1 e
	48	0.0q	0.0q	0.0q	0.0q	0.0 f
Mean		*2.6f*	*8.1e*	*10.3de*	*12.5cd*	8.4 C
General average		10.8C	16.7B	19.4A	17.1B	16.2
**	**	**				

**: significant at the 1% level; NS: no significant differences. Values followed by the same letter are not statistically significantly different. A: Presoaking duration; A: EMS application dose; a: EMS application dose × EMS exposure period interaction; a: EMS application dose × Presoaking duration; a: Three-way interaction which EMS application Dose × EMS exposure period × Presoaking duration. LSD _dose_ = 2.1; LSD _Duration_= 2.1; LSD _Presoaking_ = 2.1; LSD _Dose×Duration_=4.1; LSD _Dose×Presoaking_=4.1; LSD _Duration×Presoaking_=4.1; LSD _Dose×EMSduration×Presoaking _= 8.2; CV (%) = 37.5. CV, coefficient of variation; EMS, ethyl methanesulfonate; LSD, least significant difference.

Seedling root length is another important character of seedling stage development in rice. Seedling root length was significantly (P < 0.01) affected by EMS dose, EMS exposure period, presoaking duration, and their combinations. Seedling root length varied between 0.0–5.1 cm and the experiment average were 2.2 cm. The highest mean root length was measured at 3.5 cm for the 0% EMS dose control plot ( Table 6). The result of the seedling experiment indicated that increasing EMS doses caused a significant decrease in seedling root development. A significant decrease was observed of over 50% when EMS dose was 1% and higher. As EMS exposure period increased, a significant decrease in seedling root occurred, especially for the 24-hour EMS exposure period. At doses of 0.5% EMS and higher, an exposure period of 48 hours resulted in a significant decrease (no growth of roots) in seedling root length compared with the control. The results indicated that no presoaking caused a significant decrease in seedling root development. A significant decrease was observed of approximately 50% when seeds were not presoaked. In terms of presoaking duration, a significant decrease in seedling root length occurred especially for the 48-hour EMS exposure period. The longest seedling roots were obtained for the 12 hours presoaking duration, 2% EMS dose and six-hour exposure period conditions when compared to the other combinations.

**Table 6.** Effect of EMS application dose, EMS exposure period and presoaking duration on root length in rice seedling experiment (cm).

EMS application dose (%)	EMS exposure period (h)	Presoaking duration (h)				Mean
		0	6	12	24	
0.0%	6	4.8ab	3.9ae	3.7af	1.4il	3.5 ac
	12	3.5ag	3.1ci	3.1ci	4.1ac	3.4 ac
	24	4.0ad	4.1ac	3.3bh	3.2bh	3.6 ab
	48	2.3ej	3.9ae	4.2ac	3.3bh	3.4 ac
Mean		*3.7ab*	*3.7a*	*3.6ab*	*3.0ac*	3.5 A
0.5%	6	3.3bh	2.4dj	3.8af	3.2bh	3.1 bd
	12	0.8jl	1.5il	3.3bh	3.8af	2.3 de
	24	3.5ag	2.9ci	4.4ac	2.7ci	3.4 ac
	48	0.0l	0.0l	0.0l	0.0l	0.0 i
Mean		*1.9eg*	*1.7eg*	*2.9bd*	*2.4ce*	2.2 B
1%	6	6.66E-16	3.3bh	3.9ae	3.5ag	2.7 ce
	12	1.80E-16	3.4bh	2.9ci	2.8ci	2.3 ef
	24	0.0l	2.0gk	2.3ej	0.0l	1.1 gh
	48	0.0l	0.0l	0.0l	0.0l	0.0 i
Mean		*0.0h*	*2.2dg*	*2.3cf*	*1.6fg*	1.5 C
2%	6	2.1fk	5.1a	5.1a	4.1ac	4.1 a
	12	0.0l	0.0l	2.0gk	3.8af	1.4 fg
	24	0.0l	0.5kl	1.8hk	0.0l	0.6 hi
	48	0.0l	0.0l	0.0l	0.0l	0.0 i
Mean		*0.5h*	*1.4g*	*2.2cg*	*1.9eg*	1.5 C
General average		1.5C	2.3B	2.7A	2.2B	2.2
**	**	**				

**: significant at the 1% level; NS: no significant differences. Values followed by the same letter are not statistically significantly different. A: Presoaking duration; A: EMS application dose; a: EMS application dose × EMS exposure period interaction; *a*: EMS application dose × Presoaking duration; a: Three-way interaction which EMS application Dose × EMS exposure period × Presoaking duration. LSD _dose_ = 0.4; LSD _Duration_= 0.4; LSD _Presoaking_ = 0.4; LSD _Dose×Duration_=0.82; LSD _Dose×Presoaking_=0.82; LSD _Duration×Presoaking_=0.82; LSD _Dose×EMSduration×Presoaking _= 1.65; CV (%) = 54.5. CV, coefficient of variation; EMS, ethyl methanesulfonate; LSD, least significant difference.

Fresh seedling weight is another notable parameter that indicates seedling development after mutation. Fresh seedling weight was significantly (P < 0.01) affected by presoaking duration, EMS dose, EMS exposure period, and their combinations. Fresh seedling weight varied between 0.0-195.9 mg and the experiment average was 99.5 mg. The conclusion of the seedling experiment indicated that increasing EMS doses caused a significant decrease in fresh seedling weight. In terms of EMS dose, the highest fresh seedling weight measured was 170.2 mg for the control plot ( Table 7). A significant decrease was observed of approximately 50% when the EMS dose was 0.5% and higher. There was a significant decrease in fresh seedling weight with the increase in EMS application time, especially for the 48-hour EMS application time. It was determined that the seedlings did not develop and fresh weight was not obtained for plots with 48 hours of EMS exposure combined with EMS doses of 0.5%, 1%, and 2%. In addition, it was observed that the fresh seedling weight was dramatically decreased in the plots without presoaking. A significant decrease was observed of more than 30% when non-presoaked. In terms of presoaking duration, a significant decrease in fresh seedling weight occurred especially for the 48-hour EMS exposure period. The highest fresh seedling weight was calculated for the 12 hours presoaking duration, 0.5% EMS dose and six-hour exposure period conditions when compared to the other combinations.

**Table 7.** Effect of EMS application dose, EMS exposure period and presoaking duration on fresh seedling weight in rice seedling experiment (mg).

EMS application dose (%)	EMS exposure period (h)	Presoaking duration (hours)				Mean
		0	6	12	24	
0.0%	6	192.5ab	168.4ag	143.6ai	121.5el	156.5 bd
	12	195.9a	178.0ad	187.1ab	176.0ae	184.3 a
	24	188.5ab	168.9ag	186.1ab	163.2ai	176.7 ab
	48	150.2ai	183.4ac	165.8ah	153.2ai	163.1 ac
Mean		*181.8a*	*174.7ab*	*170.7ab*	*153.5b*	170.2 A
0.5%	6	123.3dk	110.9hm	177.5ad	118.1gm	132.4 df
	12	33.8no	81.5jn	170.7ag	175.2af	115.3 eg
	24	148.0ai	142.9ai	142.5ai	108.3im	135.4 ce
	48	0.0o	0.0o	0.0o	0.0o	0.0 i
Mean		*76.3df*	*83.8de*	*122.7c*	*100.4cd*	95.8 B
1%	6	0.0o	128.4cj	147.4ai	145.7ai	105.4 fg
	12	0.0o	150.4ai	120.6fl	138.2bi	102.3 g
	24	0.0o	68.5kn	116.4gm	0.0o	46.2 h
	48	0.0o	0.0o	0.0o	0.0o	0.0 i
Mean		*0.0g*	*86.8de*	*96.1ce*	*70.9ef*	63.5 C
2%	6	63.0mn	169.7ag	108.3im	139.3bi	120.1 eg
	12	0.0o	0.0o	66.6ln	156.0ai	55.6 h
	24	0.0o	30.0no	109.5im	0.0o	34.9 h
	48	0.0o	0.0o	0.0o	0.0o	0.0 i
Mean		*15.8g*	*49.9f*	*71.1ef*	*73.8df*	52.6 C
General average		68.4C	98.8B	115.1A	99.7B	95.5
**	**	**				

**: significant at the 1% level; NS: no significant differences. Values followed by the same letter are not statistically significantly different. A: Presoaking duration; A: EMS application dose; a: EMS application dose × EMS exposure period interaction; *a*: EMS application dose × Presoaking duration; a: Three-way interaction which EMS application Dose × EMS exposure period × Presoaking duration. LSD _dose_ = 13.8; LSD _Duration_= 13.8; LSD _Presoaking_ = 13.8; LSD _Dose×Duration_=27.7; LSD _Dose×Presoaking_=27.7; LSD _Duration×Presoaking_=27.7; LSD _Dose×EMSduration×Presoaking _= 55.3; CV (%) = 40.8. CV, coefficient of variation; EMS, ethyl methanesulfonate; LSD, least significant difference.

### EMS LD _50_ Determination

In terms of the LD _50_ dose determination study, four EMS doses were considered and other applications such as EMS exposure period and presoaking duration were ignored. EMS doses of 0%, 0.5%, 1%, and 2% were utilized in the study. In this research, LD _50_ dose was determined of surviving rates rather than germination rate. Survival rates were lower than germination rates. The Spearman-Karger method, which was introduced by Spearman in 1908 and modified by Karger in 1931, was utilized. The Spearman-Karger equation evaluated based on the dose increase rates and the number of died plants in Table 8. The highest EMS dose was 2%, and the highest EMS dose mortality rate was 85.2%. At the end of the study, 0.5% EMS dose was determined as the LD _50_ dose. When other subjects were excluded, the LD _50_ dose recommended by most researchers for mutation was 0.5% EMS dose. In this article, in which the optimum conditions of EMS application Dose, EMS exposure period, and Presoaking duration are tried to be determined, 0.5 EMS dose is recommended when evaluated together with other variables.

**Table 8.** Estimating LD _50_ lethal concentrations of EMS doses according to Spearman-Karger Method.

EMS Dose (%)	Dose Difference (a)	No of Dead (m)	Mean Mortality (b)	Product (a x b)
0	-	9.8	-	-
0.5	0.5	64.5	37.2	18.6
1	0.5	71.5	68.0	34.0
2(D _h_)	1.0	85.2	78.4	78.4
		Sum of Product		131.0
		LD _50_		0.5

LD _50_ = Arithmetic means of dose that half the sum of the plants reacting with two consecutive doses; Dh = highest dose for plants a= Half the sum of the plants reacting with two consecutive doses; b= Mean mortality of the plants between two consecutive doses; a × b = Product; m = number of died plants in each group; Sum of Product = Sum of all product; LD _50_ = Dh – [∑ (a × b) / m]. LD _50_= 2 - (131.0 / 85.2) = 0.5

## Discussion

The experimental results of both the germination experiment and the seedling experiment revealed that the presoaking duration, EMS dose, EMS exposure period, and their interactions were significant. The result of the experiment was similar to study results of Talebi *et al.* (2012), and Ramchander *et al.* (2014). Slight variations appeared in terms the most suitable combination of factors. However, results were obtained that could be used to make a standard protocol. Presoaking is an important stage for the EMS solution to diffuse into the seed and optimum presoaking duration is expressed as the presoak duration when the seed reaches full saturation. Although from previous experiments it was recommended that maximum water intake of the seeds is reached for EMS mutation application, it was determined to be useful water intake level at around 25% in this research. Rice reached this water intake level at 12 hours. The results illustrated that the rice seed reached full saturation after 24 hours presoaking. However, when the presoaking duration was evaluated on its own and with other conditions, it was determined that the 12-hour presoaking duration was the most suitable time for EMS application. In addition, EMS exposure periods of more than six hours might be damaging to the seed. The seeds might tolerate a long exposure period of 12 hours or 24 hours. However, 48 hours of application caused the seed to irreversibly lose its germination ability. EMS application doses of 0.5%, 1%, and 2% reduced surviving seeds by roughly 50%, 60%, and 80%, compared to the 0% EMS dose. The LD _50_ was determined as 0.5% EMS dose. Furthermore, the mutated seeds can be stored for three to four weeks after drying and retain more than 85% of their germination ability; the result of Tonthong *et al.* (2018) also supported this process. In this study, 12 hours presoaking duration, a six-hours EMS exposure period, and 0.5% EMS dose were determined to be the most appropriate combination. The EMS application protocol might be successfully utilized in rice mutation research.

## Conclusion

The most suitable EMS application practice was determined to be 12 hours presoaking, 0.5% EMS dose, and six hours EMS exposure for rice. The protocol includes the following: (1) Presoaking: 12 hours, (2) EMS application: 0.5% dose EMS and six hours, (3). Final washing: six hours, (4) Drying: 72 hours at 38°C. In addition, the protocol sheets are presented as a user-friendly protocol as *Extended data* ( Unan, 2021b).

## Data availability

### Underlying data

Zenodo: Dataset related paper "protocol for ems mutagenesis application in rice". https://doi.org/10.5281/zenodo.4549457 ( Unan, 2021a).

This project contains the following underlying data:

- Protocol_for_EMS_application_in_rice_data.xlsx

### Extended data

Zenodo: Factsheet related paper "protocol for ems mutagenesis application in rice".

https://doi.org/10.5281/zenodo.4587383 ( Unan, 2021b).

This project contains the following extended data:

- Factsheet of Protocol for EMS mutagenesis application in rice.pdf
- Flow Chart of Protocol for EMS Mutagenesis Application in Rice.pdf

Data are available under the terms of the Creative Commons Attribution 4.0 International license (CC-BY 4.0).
