# Supplementary material for: Protocol for ethyl methanesulphonate (EMS) mutagenesis application in rice
Source: Open Res Eur. 2022 Feb 14;1:19. Originally published 2021 Mar 24. [Version 3] doi: 10.12688/openreseurope.13317.3 (PMC10446052; doi:10.12688/openreseurope.13317.3)
Supplement: Peer review history

# Peer review: Reviewer response for version 3

**DOI:** 10.21956/openreseurope.15586.r28577

**Published:** 2022-03-29

**Authors:** Ashok Somalraju

**Affiliations:** 1 Charlottetown Research and Development Centre, AAFC, Charlottetown, PE, Canada

I am satisfied with the revisions except for the title of the research article. I am still not convinced with the explanation given by the author for generalizing it as ‘Protocol for ethyl methanesulphonate (EMS) mutagenesis application in rice’, instead of naming the specific rice variety. But that’s just a suggestion and it’s up to the author whether or not to go through with it. I am satisfied with the revisions overall and ‘APPROVE’ the manuscript in its current form.

Is the study design appropriate and does the work have academic merit?

Yes

Is the work clearly and accurately presented and does it cite the current literature?

Partly

If applicable, is the statistical analysis and its interpretation appropriate?

Partly

Are all the source data underlying the results available to ensure full reproducibility?

Yes

Are the conclusions drawn adequately supported by the results?

Yes

Are sufficient details of methods and analysis provided to allow replication by others?

Yes

Reviewer Expertise:

NA

I confirm that I have read this submission and believe that I have an appropriate level of expertise to confirm that it is of an acceptable scientific standard.

# Peer review: Reviewer response for version 2

**DOI:** 10.21956/openreseurope.15300.r27972

**Published:** 2021-11-15

**Authors:** Ashok Somalraju

**Affiliations:** 1 Charlottetown Research and Development Centre, AAFC, Charlottetown, PE, Canada

I could not find the manuscript version in Microsoft word format with line numbers listed. So, I will try to detail and list the paragraphs and sections I had issues with.

 The manuscript overall reads well. There is a proper structure and flow to the contents of the manuscript. But, there are some incomplete references in the introduction, inaccurate observations in the results section and incomplete discussion section where the observations from the results section have been shortened and duplicated as discussion.

1. My first concern is regarding the title of the manuscript. It says 'Protocol for ethyl methanesulphonate (EMS) mutagenesis application in rice'.                            There are thousands of rice varieties and the study was conducted only on *Osmancik-97. *This protocol may or may not produce similar outcomes in other rice varieties, and other rice varieties were not studied in this research. So, making the title more specific by changing it to Japonica/ *Osmancik-97 *rather than generalizing it plainly as 'rice' would be more appropriate or else would be a mischaracterization in my opinion.
                                                                               
2.  In paragraphs 3 and 4 in the introduction and in materials section, there are several statements such as, 'The main rice area, Asia, has a production system of transplanting rice so they have no severe weed problems in their fields', 'Old herbicides do not work effectively over time', 'Researchers have developed herbicide resistance systems such as Clearfield, Provisia, and Roxy Rice by mutation application', 'Rice plant breeders have used point mutations in their breeding program to overcome these problems', 'mutations exist in nature besides the artificially induced mutation', 'Physical and chemical mutagens are used to obtain plants by mutation breeding, such as gamma rays, X-rays, fast neutrons and also ethyl methanesulphonate (EMS; CH3SO3C2H5), diepoxybutane (DEB, C4H6O2) and sodium azide (NaN3)', 'The chemical mutagen EMS has been widely utilized to induce a large number of functional variations in rice', 'Osmancik-97 is a Japonica type Turkish rice variety. The variety was released by Trakya Agricultural Research Institute, Edirne, Turkey in 1997', 'The parents are Rocca and Europe, which originate from Italy'.  All these statements are used in this manuscript without any references. Please cite proper references.                                                                                                       
3. In the methods section, under 'EMS mutagenesis', it would be great if the authors could add a couple of sentences addressing how the unused and the leftover EMS solutions were neutralized and discarded, since EMS is a carcinogen. That would greatly help the future researchers that are going to follow and cite this protocol. (Just a suggestion).
                                                       
4. In the results section, under the 'Imbibition rate', In figures 1 and 2, it would be more informative to add the standard deviation/standard error bars to show case significant differences between treatments.
5. In the results section, under the 'Germination experiment', in the first paragraph it says 'Considering EMS exposure period, mean germination percentage was 91.9%, 81.3%, 55.6%, and 24.7% for six, 12, 24, and 48-hour exposure periods, respectively'. The author is getting these mean germination values after taking into account the mean of the controls (0.0% EMS) along with the rest of the EMS treatments. By doing this, the author is masking the effect of EMS treatment on germination percentage. The proper way to do this is by considering controls (0.0% EMS) as one group and the rest of the EMS treatments as the other group. This way, the authors can properly compare the mean germination values between the controls and EMS-treatments. Next sentence says  'Most combinations resulted in 100% germination'. This observation doesn’t seem accurate. Barring 0.0% EMS treatment (controls), 17 of the 48 combinations showed 100% germination whereas 31 of the 48 did not. In the next paragraph, it says 'EMS application for over 24 hours with EMS application doses of 1–2% forestalls plumula length' This observation seems inaccurate. Instead of stating EMS application for over 24 hours with EMS application, ‘EMS application for 48h with EMS application doses of 1–2% forestalls plumula length', is more accurate based on the data in table 2.
6. In the results section, under the 'Seedling experiment', In the fourth paragraph it says 'It should be emphasized that the chemical reduces the germination ability of dry seeds and also EMS application for more than 24 hours inhibit germination to a high extent'. Characterizing it as more than 24 hours is inaccurate. Since the authors have not tested any other pre-soaking times between 24 H and 48H. Calling it as ‘more than 24 hours’ might imply that pre-soaking for 25H or 26H or 30H  might inhibit germination, which may or may not be true since the authors haven’t tested that. The authors tested ‘48H’. So, it would be logical to report it as ‘EMS application for 48 hours inhibited germination to a high extent’.
       
7. In the results section, under the 'EMS LD50 Determination', it says 'In this article, in which the optimum conditions for three-way are tried to be determined, 0.5 EMS dose is recommended when evaluated together with other variables.' Three-way of what? This needs more clarity.
                                 
8. In the Discussion section, it says 'The result of the experiment was similar to study of Talebi *et al.* (2012), and Ramchander *et al.* (2014).' Please indicate how the results of this study are similar to the studies reported by Talebi *et al.* (2012), and Ramchander *et al.* (2014). The next sentence says 'Slight variations appeared in terms of the most suitable combination of factors' Combination of factors for? The sentence should either be simplified or more clarity needed. In the same paragraph, it says 'Although from previous experiments it was recommended that maximum water intake of the seeds is reached for EMS mutation application, water intake level at around 25%, reached in 12 hours, was determined to be useful in this research.' This sentence doesn’t make sense. Please rephrase it or break it down into two sentences to convey exactly what the author is trying to say. In the next line, it says 'it was determined that the 12-hour period was the most suitable time for EMS application'.12H period of pre-soaking or EMS exposure?
                                                 
9. In the discussion section, no discussion/explanation was given for why and how the increase in EMS concentrations, exposure time and pre-soaking is effecting the different factors studied in this study. What might be the physical, physiological and biochemical components of the rice seed affected by EMS-treatment/pre-soaking and how that manifests into the differences observed in terms of germination percentages, root length, shoot length etc. And if those observations are in line or not in line with other EMS studies published in rice, wheat and other food crops.                                                                                                               
  Thank you

Is the study design appropriate and does the work have academic merit?

Yes

Is the work clearly and accurately presented and does it cite the current literature?

Partly

If applicable, is the statistical analysis and its interpretation appropriate?

Partly

Are all the source data underlying the results available to ensure full reproducibility?

Yes

Are the conclusions drawn adequately supported by the results?

Yes

Are sufficient details of methods and analysis provided to allow replication by others?

Yes

Reviewer Expertise:

Plant biology, plant physiology, biochemistry, molecular biology, molecular physiology

I confirm that I have read this submission and believe that I have an appropriate level of expertise to confirm that it is of an acceptable scientific standard, however I have significant reservations, as outlined above.

# Peer review: Reviewer response for version 2

**DOI:** 10.21956/openreseurope.15300.r27776

**Published:** 2021-10-29

**Authors:** R. Kleynhans

**Affiliations:** 1 Department of Horticulture, Tshwane University of Technology, Pretoria, South Africa

No further comments

Is the study design appropriate and does the work have academic merit?

Partly

Is the work clearly and accurately presented and does it cite the current literature?

Partly

If applicable, is the statistical analysis and its interpretation appropriate?

Yes

Are all the source data underlying the results available to ensure full reproducibility?

Yes

Are the conclusions drawn adequately supported by the results?

Partly

Are sufficient details of methods and analysis provided to allow replication by others?

No

Reviewer Expertise:

NA

I confirm that I have read this submission and believe that I have an appropriate level of expertise to confirm that it is of an acceptable scientific standard.

# Peer review: Reviewer response for version 1

**DOI:** 10.21956/openreseurope.14387.r27304

**Published:** 2021-08-18

**Authors:** R. Kleynhans

**Affiliations:** 1 Department of Horticulture, Tshwane University of Technology, Pretoria, South Africa

The experiment was well designed and are testing valid factors for the induction of mutations. Data is presented with relevant factors that is usually measured when determining optimum conditions for the generation of mutations. My major concern however is that there is no indication of the actual assessment of mutations that would be present or not. Normally LD _50 _values are calculated from the measurements taken as an indication of the best doses and durations for the generation of mutations.The authors however choose a best protocol based on the best measurements for the various treatments that might not result in mutations or very low levels of mutations. I think that the data presented can easily be reworked to establish LD _50_ values that would better indicate the possible presence of mutations, especially since there is no indication of possible mutation observations given from the plants grown.

 There is also a lot of variation present in different values given for the 0% EMS control data in some of the tables, that might indicate natural variation, that is not necessarily due to treatment effects, making it imperative to rather work with LD _50_ or even LD _25_ values. Then data is presented as a percentage of the control treatments.

 The three-way interactions observed is always difficult to work with and in this case makes some of the data in the tables difficult to interpret. The indication of significant values in the tables with upper case, lower case and italic letters could be explained better. I was not always sure what is compared with what – especially with the values inside the various pre-soaking blocks. A clear explanation will help to interpret the tables with more ease. Some of the letter are also confusing – e.g. table 2 there is things like ej (letters normally follow - ef). I do not know what is compared with what when looking at all the values followed by lower case letters. Does ej actually indicate e to j (efghj)? Explaining this below the tables would make it easier to understand as well.

 Rice is one of the crops with the most induced mutations and many different protocols have been used in this regard (see review by Viana *et al* 2019). EMS has been used extensively in the past and indications are that specific doses and durations of EMS application will vary depending on cultivar used (see review by Viana *et al *2019). Selecting a general protocol is thus difficult as this might not be applicable to various cultivars or varieties. To compare results with previously published articles it is thus important to use LD _50 _values and indicate that the suggested protocol is based on these values and applicable to the tested variety. Discussion to compare data with published research can thus be improved.

 I did not pick up any other data that include the pre-soaking treatment and this could certainly be novel as there is clear indications that the pre-soaking treatment can to some extent protect the seed from the damage caused by the EMS treatment. The question still remains if this better growth values will then still result in mutations.

 The data has merit but would add better value if LD _50 _and LD _25_ values could be calculated.

 Further clarifications and questions:

- Page 4 - “Imbibition rate (%) = (last weight - first weight) × 100 / first weight”:  If this was used to describe the percentage imbibition rate - then the graphs should also reflect the % imbibition rate on the y-axis and not indicate weight in gram per 100 seed.
- Page 4: “Statistical analysis on germination showed an attendant decrease in germination with applied increases in the concentration of EMS.”  I think the whole sentence can be removed as the next sentence state the same thing and is fine with statistical proof as presented in the table.
- Page 5, para 1: Lower EMS concentrations without pre-soaking still resulted in germination. Only the higher dosages without presoaking prevented germination and all 48 hour durations prevented germination. Just correct the sentence.
- Page 6:  If I read the table correctly and you are here referring to overall pre-soaking data - the lowest value is 27.1 and not 11.2.
- Page 6: “ EMS application for over 24 hours forestalls plumula length.”  Only for 1 and 2% application. 24 hours at 0.5% still resulted in plumula growing.
- Page 7, Table 2:  If I assume correctly, this material was soaked in water to replace the EMS exposure time. There is a lot of variation in these controls with significant differences - making it difficult to interpret results further on. Is the results observed really due to the treatment or part of the natural variation? Long soaking in water (presoaking + 0% exposure also significantly changes the plumula length. These changes are however not consistent in the three-way interaction. making interpretation of other results difficult. Maybe stick to the two way interactions and single factor data.

Below is a list of corrections relating to the article:

- Intro para 1: please correct “rice producer countries” to “rice producing countries”.
- Intro para 2: please correct “particularly low and middle income nations” to “particularly for low…”
- EMS mutagenesis para 1: Please correct “using a randomized parcel design” to “using a randomized block” (correct throughout article) and “Seeds are sterilized” to “Seeds were sterilized”.
- EMS mutagenesis para 2: Please replace “Seedling survival rate is the ratio of survive seedlings” to “the ratio of surviving seedlings”.
- Factsheet and flowchart of protocol for EMS mutagenesis application in rice: Please correct ‘This flowchart shows a schematic for how..” to “This flowchart shows a schematic illustration…”
- Experiment 2: Seedling experiment – para 1: I do not think viol is the correct word in this paragraph I would rather use plant tray in all descriptions where the word viol is used.
- Experiment 2: Seedling experiment – para 1: Please replace ‘for’ with ‘with’ in the statement “The plant viols were then put in the Fitotron at 25°C and 30°C for 12-hour dark and 12-hour light cycles for 21 days”.
- Page 5, para 1: Rewrite as “ When evaluated in terms of presoaking, the lowest mean germination (49.1%) for zero hours (dry seed) presoaking.
- Table 5: Please correct “duration on shoot” to “duration on shoot length”.
- Table 6: Please correct “duration on root” to “duration on root length”.

Is the study design appropriate and does the work have academic merit?

Partly

Is the work clearly and accurately presented and does it cite the current literature?

Partly

If applicable, is the statistical analysis and its interpretation appropriate?

Yes

Are all the source data underlying the results available to ensure full reproducibility?

Yes

Are the conclusions drawn adequately supported by the results?

Partly

Are sufficient details of methods and analysis provided to allow replication by others?

No

Reviewer Expertise:

NA

I confirm that I have read this submission and believe that I have an appropriate level of expertise to confirm that it is of an acceptable scientific standard, however I have significant reservations, as outlined above.

## References

[1] : Mutagenesis in Rice: The Basis for Breeding a New Super Plant. *Front Plant Sci* .2019;10: 10.3389/fpls.2019.01326 1326 10.3389/fpls.2019.01326 31781133PMC6857675
